# Frequency of DEA 1 antigen in 1037 mongrel and PUREBREED dogs in ITALY

**DOI:** 10.1186/s12917-017-1286-y

**Published:** 2017-11-29

**Authors:** E. Carli, A. Carminato, S. Ravagnan, K. Capello, M. T. Antognoni, A. Miglio, T. Furlanello, D. Proverbio, E. Spada, A. Stefani, F. Mutinelli, M. Vascellari

**Affiliations:** 1 Canine Blood Bank, Istituto Zooprofilattico Sperimentale delle Venezie (IZSVe), Viale dell’Università 10, 35020 Legnaro, PD Italy; 2 Veterinary Laboratory “Vetlab”, via Provenza, 20 35127 Padua, Italy; 3 Department of Veterinary Medicine, Unit of Transfusion Medicine, Via S. Costanzo 4, 06126 Perugia, Italy; 4 Veterinary laboratory “San Marco”, via Sorio 114/c, 35141 Padua, Italy; 5 Department of Veterinary Medicine,Veterinary Transfusion Research Laboratory (REVLab), University of Milan, via Celoria 10, 20133 Milan, Italy

**Keywords:** DEA 1 antigen, Dog, Transfusion

## Abstract

**Background:**

The prevalence of dog erythrocyte antigen (DEA 1) in canine population is approximately 40–60%. Often data are limited to a small number of breeds and/or dogs. The aims of this study were to evaluate frequency of DEA 1 in a large population of purebred and mongrel dogs including Italian native breeds and to recognize a possible association between DEA 1 and breed, sex, and genetic and phenotypical/functional classifications of breeds. Frequencies of DEA 1 blood group collected from screened/enrolled blood donors and from healthy and sick dogs were retrospectively evaluated. The breed and the sex were recorded when available. DEA 1 blood typing was assessed by immunocromatographic test on K3EDTA blood samples. The prevalence of DEA 1 antigen was statistically related to breed, gender, Fédération Cynologique Internationale (FCI) and genotypic grouping.

**Results:**

Sixty-two per cent dogs resulted DEA 1+ and 38% DEA 1-. DEA 1- was statistically associated with Dogo Argentino, Dobermann, German Shepherd, Boxer, Corso dogs, the molossian dogs, the FCI group 1, 2 and 3 and the genetic groups “working dogs” and “mastiff”. DEA 1+ was statistically associated with Rottweiler, Briquet Griffon Vendéen, Bernese mountain dog, Golden Retriever, the hunting breeds, the FCI group 4, 6, 7 and 8 and the genetic groups “scent hounds” and “retrievers”. No gender association was observed.

**Conclusions:**

Data obtained by this work may be clinically useful to drive blood donor enrollment and selection among different breeds.

## Background

Blood groups are defined according to antigenic and species-specific genetic markers localized on the surface of erythrocyte cell membranes [1, 2]. Canine blood groups have received international standardization and were classified using the acronym DEA (dog erythrocyte antigen) followed by a number [2–5]. Historically, DEA 1 system included the types DEA 1.1 and DEA 1.2 (and possibly DEA 1.3) [2, 5]. Virtually, all DEAs can induce alloantibody formation but the most immunogenic antigen seemed to be DEA 1.1 [2, 4]. Recently, the DEA 1 blood group system has been described as a complex autosomal dominant allelic system with varied surface antigen expression levels [7–9]. Then, a dog could be classified as DEA 1 negative (DEA 1-) or DEA 1 positive (DEA 1+) with weak to strong antigen expression [7, 8]. The proportion of DEA 1- and strong DEA 1+ dogs were recently reported to be far larger than those of weak and moderate DEA 1+ dogs [9].

Antigens other than DEA were discovered in dogs, i.e. *Dal* [10–12], Kai 1 and Kai 2 [9].

The canine blood typing for the red blood cell antigens, other than DEA 1, can be done only by a laboratory. In clinical practice, for in-house canine blood group DEA 1 determination, commercial point-of-care typing tests i.e. typing cards and immunochromatographic strips, using murine monoclonal anti-DEA 1 antibodies, are available [7, 13–15].

The general prevalence of DEA 1 in canine population is approximately 40–60% [9, 16–18]. Information about frequency of DEA 1+ and DEA 1- blood group between breeds is available but often involved a limited number of breeds. Data have been reported in Japan [19], Brazil [17, 20], South Africa [21], Nigeria [22], Croatia [23, 24], US [16, 25], Portugal [26], Turkey [6], Spain [18], India [27], Switzerland [28], Romania [29] and North America [9].

The aims of this study were (1) to evaluate frequency of DEA 1 in a large population of purebred and mongrel dogs including Italian native breeds and (2) to recognize a possible association between DEA 1+ and DEA 1- blood groups and breed, sex and genetic and phenotypical/functional breed grouping.

## Methods

### Dogs, samples and DEA 1 antigen assessment

This is a cross-sectional study without an “a priori” definition of the sample. It includes data collected from January 2013 to December 2015 and obtained from dogs screened and/or enrolled as blood donors and from healthy or sick dogs evaluated during clinical activity. DEA 1 blood typing and breed were available in all the dogs, and sex in the majority of the cases. Data were obtained from the Canine Blood Bank at the Istituto Zooprofilattico Sperimentale delle Venezie (Padua, Italy) (*n* = 354), the Department of Veterinary Medicine, Unit of Transfusion Medicine at the University of Perugia (Italy) (*n* = 313), the Veterinary Laboratory “San Marco” (Padua, Italy) (*n* = 242) and the Department of Veterinary Medicine at the University of Milan (Italy) (*n* = 128).

Blood was collected from cephalic or jugular vein into K3EDTA tubes, stored at 4–6 °C and processed within 24 h of collection. DEA 1 blood group was determined using an immunochromatographic strip typing kit (Labtest DEA 1, Alvedia, Limonest, France) according to the manufacturer’s instructions. Based on the test results, the dogs were classified as DEA 1+ and DEA 1–. For DEA 1+ dogs the band strength was not assessed.

### Statistical analysis

The differences of DEA 1 prevalence in relation to sex and breed were tested by chi-square test or Fisher’s exact test when appropriate. Data of the breeds represented by at least 15 dogs were used for breed statistical analysis referred to frequencies. Moreover for breed comparison, dogs were grouped following different criteria: i) based on Fédération Cynologique Internationale (FCI, http://www.fci.be/en/) breed classification (10 groups); ii) based on genetic grouping (10 groups) as reported by vonHoldt et al. (2010); iii) breeds with hunting function included in FCI group 6 (Scent hounds and related breeds), 7 (Pointing dog) and 8 (Retrievers-Flushing dogs-Water dogs) compared to non-hunting breeds; iv) molossian dogs belonging to both FCI group 2 (Pincher and Schnauzer-Molossoid breeds-Swiss Mountain and Cattle dogs) and group 9 (Companion and Toy dogs) compared to non-molossian dogs.

The Italian Maremma hound breed was not classified in a FCI group but it was considered as a hunting breed for statistical analysis.

Chi-square value, *P* value, odds ratios (OR) and their 95% confidence interval (95% CI) were calculated for each comparison made. In case of zero value, a unity was added to each number in the calculation of the ORs. The mongrels group was chosen as the reference group. For data concerning FCI and genetic group classification, every group was compared with the others and not with the mixed breed dogs.

Statistical analyses were performed using the freely available online software WinEpi 2.0 (http://www.winepi.net/uk/index.htm) [30]. Values of *p* < 0.01 were considered significant.

## Results

In the present study, 1037 dogs were evaluated. They were mongrels (*n* = 205) and purebred (*n* = 832), male (*n* = 498) and female (*n* = 523). The sex was unknown for 16 dogs. Purebred dogs belonged to 88 breeds. Thirty-eight percent (396/1037) of all the dogs tested DEA 1- and 62% (641/1037) DEA 1+. The percentages remained the same considering the mongrel and purebred subgroups. Dogs belonging to 14 breeds were statistically analyzed. In Tables 1 and 2 the frequencies of DEA 1- and DEA 1+ dogs according to breed and subjected to statistical analysis were summarized and related to data available in literature (when referred at least to 15 dogs per breed). In Table 3 were reported the frequencies related to breeds not subjected to statistical analysis. DEA 1- blood group was observed in all the Dogo Argentino and in mostly Dobermann, German Shepherd, Boxer and Corso dogs. All Rottweiler, Dachshound, Italian Maremma hound, Grand bleu de Gascogne, Anglo-français de petit vénierie and mostly Briquet Griffon Vendéen, Bernese mountain dog, English Setter, Giura hound, Golden Retriever, Labrador Retriever, Pinscher/Zwergpinscher and Ariégeois dogs resulted DEA 1 + .

**Table 1:** Frequency of DEA 1– blood type in breeds including15 dogs, statistical analysis results and summary of published data**

Breed	n° of dogs	% DEA 1-	*P* value	Chi-square	OR	95% CI for OR	% DEA 1- and location in literature
Dogo Argentino	56	100	<0.01*		93.08	12.63–686.15	95 B1
Dobermann	17	94	< 0.01	20.63	26.60	3.46–204.55	Data referred to groups <15 dogs
German Shepherds	65	92	< 0.01	59.18	19.95	7.67–51.84	90 B1; 84 SA; 30 I; 0 B2
Boxer	22	82	< 0.01	15.99	7.48	2.44–22.92	Data referred to groups <15 dogs
Corso dog	31	68	<0.01	10.10	3.49	1.56–7.80	71 It

*calculated by Fisher’s exact test, **only data obtained from breed with ≥15 dogs were reported

*B1* Brazil, [17]; *B2* Brazil, [20]; *I* India, [27]; *It* Italy [38]; *J* Japan, [19]; *P* Portugal, [26]; *S* Switzerland, [28]; *SA* South Africa: [21]

**Table 2:** Frequency of DEA 1+ blood type in breeds including at least 15 dogs, statistical analysis results and summary of published data**

Breed	n° of dogs	% DEA 1+	*P* value	Chi-square	OR	95% CI for OR	% DEA 1+ and location in literature
Rottweiler	15	100	<0.01*		9.02	1.17–69.67	100 B1; 88P; 86 B2; 78 SA
Bernese Mountain dog	50	94	< 0.01	18.6	9.4	2.84–31.32	100 S
Briquet Griffon Vendéen	32	94	< 0.01	12.21	9.02	2.10–38.82	None
English setter	21	86	0.03	4.51	3.61	1.03–12.65	53 J
Golden Retriever	77	83	< 0.01	11.01	2.96	1.53–5.73	95 B1
Labrador Retriever	69	77	0.03	4.76	1.99	1.06–3.73	87 I; 55 SA; 45 P
Ariegeois	55	73	0.16	2.01	1.60	0.83–3.09	None
Newfoundland	21	62	0.96	0.02	0.98	0.39–2.46	None
Italian short/rough-haired hound	16	62	0.99	0	1.00	0.35–2.87	None

*calculated by Fisher’s exact test; **only data obtained from breed with ≥15 dogs were reported

*B1* Brazil, [17]; *B2* Brazil, [20]; *I* India, [27]; *J* Japan, [19]; *P* Portugal, [26]; *S* Switzerland, [28]; *SA* South Africa: [21]

**Table 3:** Frequency of DEA 1+ and DEA 1 – blood types in breeds not statistically analyzed

Breed	Dogs (n)	DEA 1+ (%)	DEA 1– (%)
Cocker Spaniel	14	57	43
Border Collie	13	38	62
Anglo français de petite venerie	12	100	0
Giura Hound	12	83	17
Pinscher/Zwergpinscher	12	75	25
Dachshound	11	100	0
Jack Russel Terrier	10	50	50
Italian pointing dog	8	75	25
Gran Bleu de Gascogne	8	100	0
Italian Maremma hound	8	100	0
Yorkshire Terrier	8	68	32
Poodle	7	100	0
Beagle	7	71	29
Kurzhaar	7	86	14
Bullmastiff	7	71	29
Weimaraner	7	71	29
American Staffordshire	5	20	80
Bouledogue francais	5	20	80
Great dane	5	80	20
Cavalier King Charles spaniel	5	80	20
Epagneul Breton	5	100	0
Maremma and Abruzzes sheepdog	5	100	0
Posavatz hound	5	100	0
English Bulldog	4	0	100
West Highland White Terrier	4	25	75
Pug	4	100	0
Maltese	4	75	25
Italian pointer	4	100	0
Rhodesian Ridgeback	4	100	0
Pomeranian Spitz/Volpino italiano	4	75	25
Other prevalently DEA 1+ *	49	86	14
Other prevalently DEA 1-**	22	8	92

*Afgan hound, Akita inu, Alaskan Malamute, Anatolian shepherd, Basset hound, Bavarian hound, Beauceron, Bergamasco shepherd, Bolognese, Caucasian shepherd, Chihuahua, Clumber spaniel, Dalmatian, Galgo espagnol, Irish setter, Italian greyhound, Korthal, Langharr, Leonberger, Petit bleu de Gascogne, Romagna water dog, Saint Bernard dog, Samoiedo, Schnauzer, Shar-pei, Shiba inu, Spinone, Springer spaniel

**Bichon a poil frise, Bichon Havanas, Black and tan coonhound, Bobtail, Boston terrier, Bull terrier, Dogue de Bordeaux, Drahataar, Flat coated, Fox terrier, Greyhound, Saluki, Scottish shepherd, Shitzu, Siberian Husky, Black russian terrier

No difference in the prevalence of DEA 1 antigen was observed in relation to gender (p: 0.21, chi- square: 1.58, OR: 0.843, 95% CI: 0.65–1.09).

Data about DEA 1 frequency of dogs (*n* = 79) belonging to an Italian breeds are reported in Tables 1, 2 and 3.

The hunting dogs (*n* = 392, breeds *n* = 33) resulted statistically associated with DEA 1+ (81%; *p* < 0.01, chi- square: 121.92, OR: 5.54, 95% CI: 4.04–7.60). The molossian dogs (*n* = 158, breeds *n* = 16) resulted statistically associated with DEA 1- (67%; *p* < 0.01, chi-square: 68.18, OR: 4.41, 95% CI: 3.05–6.38).

All purebred dogs were classified based on FCI groups and dogs belonging to 42 breeds (*n* = 468) were classified according to the genetic groups proposed by vonHoldt et al. (2010). Based on FCI classification dogs belonging to groups 1 “Sheepdogs and cattledogs, except swiss cattledogs” (72/89, 81%), 2 “Pinscher and Schnauzer-Molossoid and swiss mountain and cattledogs” (136/254, 53%) and 3 “Terriers” (18/30, 60%) were more likely to be DEA 1- (p < 0.01). Dogs belonging to groups 4 “Dachshunds” (11/11, 100%), 6 “Scent hounds and related breeds” (129/157, 82%), 7 “Pointing dogs” (51/60, 85%), and 8 “Retrievers – Flushing dogs – Water dogs” (131/167, 78%) were more likely DEA 1+ (p < 0.01). Dogs belonging to genetic groups “working dogs” (77/91, 85%) and “mastiff-like” (36/51, 71%) were significantly more likely to be DEA 1- (p < 0.01) while dogs belonging to genetic groups “scent hounds” (47/51, 92%) and “retrievers” (152/191, 80%) were significantly more likely to be DEA 1+ (p < 0.01).

## Discussion

The present study reports data about DEA 1 frequency obtained from a large canine population including purebred dogs belonging to 88 breeds and tested with a commercial immunochromatographic kit. Few reports are available on breed-specific DEA 1 frequencies, often involving a limited number of dogs and breeds. They were reported in Japan [19], Brazil [17, 20], South Africa [21], Portugal [26], Spain [18, 31, 32], Switzerland [28] and India [27]. A large survey of dogs has been performed in the US but the detailed results were not published [17].

The DEA 1 antigen was present in the 62% of the dogs included in this study without difference between mongrel and purebred subgroups. In agreement with our results, canine population frequencies range between 53% in Switzerland [28] and 61% in Brazil [17]. In contrast, lower percentages are reported in US (42%) [16] and Japan (44%) [19]. In mongrels, inhomogeneous results are published, with DEA 1+ dogs ranging from 48 [21] to 90% [20].

Considering breed-specific frequency results, DEA 1- group was statistically associated with Dogo Argentino, Dobermann, German shepherd, Boxer and Corso dogs. Frequencies obtained agree with those previously reported (Table 1). Only for German Shepherd dogs frequencies of 100% and 70% of DEA 1+ were reported in Brazil [20] and India [27] respectively.

DEA 1+ blood group resulted statistically associated with Rottweiler, Briquet Griffon Vendéen, Bernese mountain dog and Golden Retriever breeds. Moreover dogs belonging to English setter, Labrador Retriever, and Ariégeois resulted prevalently DEA 1+ with frequencies ranging from 73 to 94%. Our results were in agreement with data reported elsewhere for Golden retriever [17] and Bernese mountain dog [28]. Our Rottweiler were 100% DEA 1+ as reported in Brazil [17], even if elsewhere 12–22% of DEA 1- dogs were described [20, 21]. To the best of our knowledge, this is the first data about DEA 1 frequency for Briquet Griffon Vendéen, and Ariégeois. More extensive surveys would be strongly required to confirm the results of the present work, especially for breeds represented by a low number of dogs.

The discrepancies observed in our frequencies referred to mongrel and some purebred dogs compared to other studies could be related to the geographical different expression of the antigen as previously hypothesized [26, 33]. The performance of the diverse DEA 1 blood typing tests used in the different works published worldwide, may be also considered as a possible explanation. Even though no currently accepted gold standard for DEA 1 blood typing is available [14], the kit used in the present work seems to have the better performance. In fact when compared with other methods (i.e. card agglutination assay or gel-based method), it resulted specific and less subjective than the sensitive typing cards [15]. Moreover, recent flow cytometric typing studies demonstrated a significant correlation with chromatographic strips for DEA 1 assessment, using the same monoclonal anti DEA 1 antibody [7, 8]. A possible limitation of the present study may be that only one blood typing method was used both in healthy and sick animals. Since it is a retrospective study, no information about the presence and the severity of anemia of ill dogs are available. The reduction of PCV has been reported to cause a decrease in the intensity of the DEA 1 band. [15, 34]. Consequently, in our survey a limited number of false negative results could not be excluded. Studies including dogs of different geographical area, obtained with the same test and, possibly, with the band straight assessment should be helpful to reduce influences (i.e few breeders, dogs closely related, test performance) on DEA 1 frequency referred to breed.

The FCI nomenclature splits purebred dog into ten groups based on morphology, current use and historical criteria. Recently, genetic diversity of dog breeds was evaluated and surprisingly a correspondence between genetic and phenotypical/functional breed grouping was observed with some exception [35]. Dogs were accurately assigned to their breed >99% of the time [35] and the various breeds related one to another [35]. Related groups of breed were defined by these studies and a correlation with breed genetic relatedness, morphological features and geographic origin was observed [36, 37]. Dogs belonging to our database were classified based on FCI groups and genetic groups involving breeds that clustered together as previously reported [35]. Remarkably dogs belonging to FCI groups 1 and 2 resulted statistically more likely to be DEA 1- and dogs belonging to groups 4, 6, 7, 8 resulted more likely to be DEA 1+. In addition dogs belonging to genetic groups “working dogs” and “mastiff-like” resulted more likely DEA 1- and the groups “scent hounds” and “retriever” were more likely DEA 1+ .

Furthermore, molossoid dogs resulted statistically associated to DEA 1- blood group while dogs belonging to hunting breeds were significantly associated with DEA 1+ blood group. More data have to be collected to confirm this observation.

Frequency of only DEA 1 antigen was studied in the present work. Extended canine blood groups assessment including DEA other than 1 should be performed on candidate blood donor but it was not easily available for clinicians. In the clinical practice only DEA 1 kits are commercially available and could be used before the transfusion to test donor and recipient. Consequently the resulted reported in the present large canine population study could be exploited by clinicians to choose the most suitable dog to test in order to find the right candidate blood donor quickly.

## Conclusion

The present study provides data on the frequency of DEA 1 antigen in a large number of dogs and confirms a broad variability among breeds. In fact, some breeds show high frequency of DEA 1- subjects resulting more susceptible to immunization when they receive blood from an incompatible donor.

In the routine activity of clinicians and of canine blood bank, our results may be valuable to drive blood donor enrollment and selection among different breeds.

## Abbreviations

95% CI: 95% confidence interval
DEA: Dog erythrocytes antigen.
FCI: Fédération Cynologique Internationale.
OR: Odds ratios.
